# Barriers and Facilitators to Delirium Management Among ICU Nurses in Two Tertiary Hospitals in Chongqing, China: A Qualitative Study Using the COM-B Framework

**DOI:** 10.3390/healthcare14162646

**Published:** 2026-08-20

**Authors:** Yulong Cao, Yan Gao, Ruiqi Yang, Xueyuan Luo, Xinyi Liu, Xiaoxiao Wei, Xiuni Gan, Xiaomin Sheng

**Affiliations:** 1 Department of Nursing, The Second Affiliated Hospital of Chongqing Medical University, Chongqing 400010, China; 2024122224@stu.cqmu.edu.cn (Y.C.);; 2 Intensive Care Unit, The Second Affiliated Hospital of Chongqing Medical University, Chongqing 400010, China

**Keywords:** delirium, intensive care units, nurses, qualitative research, behaviour change, COM-B

## Abstract

**Background:** Delirium management remains inconsistently implemented in intensive care units (ICUs), and existing studies provide limited insight into how assessment capability, organisational opportunity, and emotional motivation interact in Chinese tertiary care settings. Aim: To explore ICU nurses’ perceived barriers and facilitators to delirium assessment and management using the Capability, Opportunity and Motivation model of Behaviour (COM-B). **Methods:** A qualitative descriptive exploratory study was conducted with 14 registered ICU nurses from two tertiary hospitals in Chongqing, China, between December 2025 and February 2026. Purposive sampling captured variation in clinical experience, educational background, and ICU type. The first author conducted face-to-face interviews in Mandarin Chinese and completed the initial coding. COM-B informed both the interview guide and the deductive organisation of the analysis, while six themes and 33 lowest-level factors were developed within the three domains. During revision, the fixed analytic structure was audited against all 14 Mandarin transcripts, and the second author independently applied the fixed codebook to five complete transcripts selected for maximum variation; disagreements were resolved by returning to the original Mandarin context and predefined coding boundaries. **Results**: Eighteen barriers and fifteen facilitators were identified across six themes within the COM-B domains. Capability was strengthened by clinical experience, senior nurses’ intuitive pattern recognition, and conversational assessment, but constrained by difficulty recognising hypoactive delirium, uncertainty when assessing older adults, inconsistent use of CAM-ICU, and insufficient targeted training. Opportunity was enhanced by staffing, environmental control, peer support, family engagement, and physician collaboration, but restricted by fragmented visibility, sensory overstimulation, workload, restrictive visiting, and delayed medical responses. Motivation reflected the tension between professional duty and accountability for safety on one hand and emotional burden, verbal abuse, and violence on the other. Cross-domain accounts showed that opportunity constraints could amplify anxiety and safety-first motivation, narrowing feasible behaviour toward containment, whereas social support could sustain relational care. **Conclusions**: Delirium management was shaped by interacting behavioural, organisational, and emotional conditions. Strengthening assessment capability alone is unlikely to produce consistent practice without staffing, workflow, environmental, and interprofessional support. Mapping the findings to Behaviour Change Wheel intervention functions highlighted training, environmental restructuring, enablement, and modelling as priority candidate functions for prospective evaluation; education alone was unlikely to be sufficient. **Reporting Method**: This study was reported in accordance with the Consolidated Criteria for Reporting Qualitative Research checklist. **Patient or Public Contribution**: No patient or public contribution was involved in the design, conduct or reporting of this study. Participants were registered intensive care unit nurses.

## 1. Introduction

Delirium is a common and serious complication of critical illness, characterised by acute, fluctuating disturbances in attention, awareness, and cognition [1]. In intensive care units (ICUs), delirium affects both mechanically ventilated and non-ventilated patients, and it is often under-recognised, particularly when symptoms are subtle or hypoactive [2]. The clinical impact of delirium extends beyond transient behavioural disturbance. It is associated with longer durations of mechanical ventilation and ICU stay, more complications, higher mortality, and persistent neurocognitive impairment after hospital discharge [3,4]. Collectively, these adverse outcomes make delirium a major contributor to the long-term survivorship burden after critical illness [5].

Accordingly, contemporary ICU practice treats systematic delirium prevention, detection, and management as core elements of high-quality care [6]. International guidelines and care bundles recommend routine delirium monitoring with validated instruments, optimising analgesia and sedation, promoting early mobilisation and sleep, supporting reorientation, and reducing modifiable risk factors [7,8]. Although delirium management is multidisciplinary, ICU nurses are central to delivery because they provide continuous bedside surveillance, conduct repeated assessments, and implement first-line non-pharmacological strategies in real time [9]. Nurses also escalate concerns to medical staff when delirium is suspected, balance competing priorities during clinical deterioration, and involve families to reinforce reorientation and reduce distress. Therefore, the effectiveness of delirium care pathways depends on nursing practice and the clinical context in which care is delivered.

Despite a strong evidence base and extensive guideline dissemination, delirium management is implemented inconsistently across ICUs. Delirium screening is unevenly integrated into routine workflows, and preventive strategies are often deprioritised when workload, staffing constraints, and acute physiological instability dominate bedside decision-making [10,11,12]. Even when assessment tools are available, uptake may be limited by perceived lack of feasibility, frequent interruptions, and uncertainty in interpreting responses in patients who are sedated, have communication barriers, or have baseline cognitive impairment [13]. Consequently, delirium care may become episodic and reactive, initiated mainly in response to overt agitation rather than proactive recognition and prevention; this implementation gap persists across many health systems [12].

Recent reviews and theory-informed studies have already identified nurse-level, organisational, and implementation barriers to delirium care [12,14]. The remaining gap is therefore not whether such barriers exist, but how they interact within particular clinical contexts and shape bedside decisions. Evidence is especially limited from Chinese tertiary ICUs, where unit design, staffing patterns, family access, and interprofessional arrangements may influence nurses’ ability to sustain relational and non-pharmacological care. Emotional labour arising from agitation, verbal abuse, and occupational violence has also received comparatively little attention as a determinant of nurses’ motivation.

The Capability, Opportunity and Motivation model of Behaviour (COM-B) provides a pragmatic framework for examining these interacting determinants [15]. COM-B proposes that behaviour occurs through the combined influence of capability, opportunity, and motivation. In the present study, the Chongqing setting enabled examination of how assessment competence, unit-level and social conditions, safety-oriented reasoning, and emotional endurance intersected across comprehensive, emergency, and respiratory ICUs. Accordingly, this study aimed to identify ICU nurses’ perceived barriers and facilitators to delirium assessment and management using COM-B, with particular attention to the contribution of the Chinese tertiary-hospital context, emotional labour, and restricted family presence.

## 2. Methods

### 2.1. Study Design

A qualitative descriptive exploratory design was used to provide a close, practice-oriented account of the barriers and facilitators experienced by ICU nurses in delirium management [16,17]. The findings were generated through COM-B-guided thematic analysis rather than a standalone framework analysis: COM-B supplied the three deductive organising domains, whereas the six experiential themes and the lowest-level barriers and facilitators were developed from the interview data. Reporting was guided by the Consolidated Criteria for Reporting Qualitative Research (COREQ) checklist [18] (Supplementary File S1).

### 2.2. Theoretical Framework

COM-B informed both data collection and analysis [15]. The interview guide used prompts addressing capability (knowledge and practical skills for recognition, assessment, and management), opportunity (workload, time, staffing, team collaboration, physical environment, and family involvement), and motivation (safety-oriented reasoning, professional responsibility, emotional responses, and beliefs about recovery). During analysis, capability, opportunity, and motivation were treated as predefined domains. Themes and their constituent barriers and facilitators were then developed within these domains, allowing the framework to organise the findings without predetermining their specific content. After the thematic structure had been finalised and verified, selected determinants were mapped to Behaviour Change Wheel intervention functions [15] as an interpretive step to generate candidate practice strategies; this mapping did not contribute to theme generation and should not be interpreted as intervention testing.

### 2.3. Setting and Sample

The study was conducted in ICUs at two tertiary hospitals in Chongqing, China. Eleven participants were recruited from Site 1 and three from Site 2; eight worked in comprehensive ICUs, three in emergency ICUs, and three in respiratory ICUs. Eligible participants were registered nurses who had independently provided bedside care for more than one year. Trainee nurses, nurses without independent bedside responsibilities, nursing managers without current bedside roles, and non-nursing professionals were excluded. Purposive sampling sought variation in ICU experience, education, professional role, and ICU type. The two sites were used to broaden contextual variation rather than as separate comparative samples; the study was therefore not designed to support site-specific thematic saturation or formal between-site comparisons.

At Site 1, RY, an ICU nurse and research team member, circulated study information and facilitated initial contact with potential participants; she was a colleague of some participants but did not conduct interviews, transcribe data, or undertake coding. At Site 2, XS facilitated access and had no employment or supervisory relationship with participants. XG held a senior nursing administrative position at Site 1 but was not the participants’ immediate clinical supervisor and was not informed of individual decisions to participate or decline. YC and YG had no prior working relationship with participants. The number of eligible nurses approached or declining participation was not formally recorded; no participant withdrew after consent.

### 2.4. Data Collection

All 14 interviews were conducted in person and in Mandarin Chinese by the first author (YC) in a private room at the participants’ workplaces. Interviews were scheduled at participants’ discretion during a lunch break or after an afternoon shift; no managers, colleagues, or other non-participants were present. No interviews were interrupted. YC had prior experience in qualitative data collection and analysis through participation in an earlier study of ICU visitation, but was not a specialist in delirium research. Interviews lasted 24–37 min, were audio-recorded, and were transcribed verbatim by YC [19]. The semi-structured interview guide (Supplementary File S2) was developed from COM-B. Three nurses with clinical backgrounds similar to those of the study participants completed pilot interviews, after which the guide was revised. The pilot data were not coded or included in the final analysis. No repeat interviews or field notes were undertaken.

Data collection and preliminary analysis proceeded concurrently. Data adequacy was judged across the combined purposive sample rather than separately within each hospital. By the 12th interview, no substantively new codes or themes were identified, and two additional interviews were conducted to check whether the developing thematic structure remained adequate, resulting in a final sample of 14. No formal saturation log was maintained, and the unequal site contribution (11 participants from Site 1 and three from Site 2) was not interpreted as evidence of site-specific saturation. YC and YG reviewed the emerging coding structure and interpretive issues during this period. Transcripts were not returned to participants, and participant checking was not undertaken.

### 2.5. Data Analysis

The Mandarin transcripts were analysed in NVivo 12 using the six phases of thematic analysis described by Braun and Clarke [20]. YC completed the initial coding. Capability, opportunity, and motivation were applied deductively as organising domains, while the six themes and the factors within them were developed from the interview data. Each lowest-level factor was given a distinct operational meaning so that closely related concepts, such as staff shortage versus high workload and professional duty versus accountability, could be differentiated. During revision, two complementary verification procedures were undertaken without re-generating the analytic framework. First, a full-dataset fixed-codebook concordance audit checked all 33 factors against all 14 Mandarin transcripts, including direct support, boundary cases, and contrasting evidence (Supplementary File S3). Second, YG independently applied the same fixed 33-factor codebook to five complete transcripts (P02, P04, P09, P12, and P14) purposively selected to span both hospitals, general/EICU/RICU settings, junior-to-senior experience, and bachelor’s/master’s preparation. YG completed first-pass coding before viewing YC’s transcript-level assignments. At the participant-by-factor presence/absence level, 137 of 165 decisions were concordant (83.0%; descriptive Cohen’s κ = 0.656), leaving 28 disagreements. For each disagreement, YC and YG returned to the original Mandarin passage and surrounding context, reviewed the prespecified operational definition and inclusion/exclusion boundary, and discussed the interpretation until consensus was reached; neither coder’s initial assignment was treated as the default. Ten disagreements retained the YC/full-audit interpretation and 18 retained the YG interpretation. Reconciliation refined participant-level factor assignments but did not alter the six-theme, 18-barrier/15-facilitator structure (Supplementary File S4).

All coding, audit decisions, and consensus discussions were conducted using the Mandarin source text. Selected quotations were translated into English only after the Chinese analysis was complete. YC prepared the English renderings and YG compared them with the original Mandarin passages and surrounding context for conceptual equivalence, with particular attention to emotionally nuanced expressions. Formal independent back-translation was not undertaken.

#### Researcher Reflexivity and Trustworthiness

Recruitment relationships were treated as a potential source of influence rather than as a neutral procedural detail. At Site 1, RY was a colleague of some participants and facilitated initial contact, which could have affected who volunteered or how safe participants felt discussing workplace practice. To separate recruitment from data generation, RY did not conduct interviews or coding; all interviews were conducted privately by YC, who had no prior working relationship with participants, and no managers, colleagues, or other non-participants were present. XG’s senior administrative role was also considered potentially influential, although she was not the participants’ immediate supervisor and was not informed of individual participation decisions. These safeguards reduced, but could not eliminate, selection and social-desirability effects. YC’s previous involvement in qualitative research on ICU visitation was additionally treated as a sensitising influence, particularly for family-related accounts; these interpretations were discussed with YG and checked against the complete dataset, including limiting or contrasting accounts.

Credibility was supported through purposive variation in experience, role, education, and ICU type; concurrent data collection and preliminary analysis; repeated reading of transcripts; comparison across participants, hospitals, and ICU types; and two additional interviews after no substantively new codes or themes were identified. Transcript return and participant checking were not used as formal validation strategies. Instead, the cross-case interpretations were challenged against divergent accounts and linked back to identifiable Mandarin transcript evidence, supplemented during revision by a full-dataset fixed-codebook audit and independent subset coding.

Dependability and confirmability were strengthened by explicit operational definitions and inclusion/exclusion boundaries for all 33 factors, preservation of boundary and contradictory evidence, and a documented YC–YG disagreement-resolution trail. The revision-stage subset check was used to test the application of the fixed framework rather than to claim that qualitative interpretation can be reduced to a single reliability coefficient; the reported κ is therefore a descriptive audit indicator at the participant-by-factor level. Transferability was addressed by detailed reporting of site, ICU type, participant characteristics, recruitment relationships, and analytic procedures.

### 2.6. Ethical Considerations

Ethical approval was obtained from the Ethics Committee of the Second Affiliated Hospital of Chongqing Medical University (Approval No. 2025-296) before data collection, and institutional permission was obtained from both hospitals. Written informed consent was obtained privately by YC. Participants were explicitly informed that participation was voluntary; declining or withdrawing would not affect employment, appraisal, education, or professional relationships; and individual participation decisions would not be disclosed to nursing managers. All data were anonymised, securely stored, and handled confidentially.

## 3. Results

### 3.1. Participant Characteristics

Fourteen ICU nurses participated (mean age 32.8 years, SD = 5.1, range = 25–43; mean ICU experience 8.6 years, SD = 4.5, range = 2–18). Ten participants were women and four were men. Eleven participants were from Site 1 and three from Site 2; eight worked in comprehensive ICUs, three in emergency ICUs, and three in respiratory ICUs. All held at least a bachelor’s degree, and three held a master’s degree. Twelve reported previous delirium-related training, whereas four reported that their unit had a standardised delirium management protocol. No participant withdrew after consent. The number of eligible nurses approached or declining participation was not formally recorded (Table 1).

### 3.2. COM-B Domains and Themes

Three COM-B domains contained six inductively developed themes and 33 lowest-level factors: 18 barriers and 15 facilitators (Table 2). All six themes were supported by accounts from multiple participants and more than one ICU type.

### 3.3. Capability

#### 3.3.1. Navigating the Complexities of Assessment

Thirteen participants described hyperactive delirium as comparatively easy to identify because agitation, disorganised speech, and unsafe behaviours were directly observable. Eight participants described hypoactive or “quiet” delirium as subtle and liable to be missed unless nurses deliberately initiated interaction, while six described conversational assessment as a useful supplement to formal screening. One participant explained:

*“Actually, delirium has a manic type… and also a quiet type. The quiet type is not easy to detect, so we need to actively chat to judge if they answer questions incorrectly.”*

(P13)

Seven participants described practical difficulties in applying standardised assessment tools, particularly CAM-ICU, when patients were unable or unwilling to engage. Two specifically highlighted uncertainty when assessing older adults, for whom limited comprehension or baseline cognitive impairment made it difficult to distinguish acute inattention from pre-existing limitations. One nurse reported:

*“Especially some elderly patients have limitations in knowledge and comprehension… They often give irrelevant answers, and you cannot tell whether they simply do not know or are responding incoherently due to delirium, so assessment is quite difficult.”*

(P04)

The seven participants who described difficulty or limited routine use of formal tools did not uniformly abandon assessment. Instead, they supplemented structured tools with simplified conversational questions, observation of fluctuation, and comparison with the patient’s previous cognitive state.

#### 3.3.2. Perception of Clinical Experience and Training

Seven participants described clinical experience as strengthening early recognition and management. Three specifically emphasised senior nurses’ intuitive judgement and timely support, while four described subtle behavioural or interactional cues that became more recognisable with experience. Three participants contrasted novice with experienced nurses, reporting that less experienced staff were more likely to recognise delirium only after overt agitation or unsafe behaviour had emerged. One participant noted:

*“There is a significant disparity between new and experienced nurses in their ability to manage delirium. The department tends to assign new nurses to night shifts; however, these nurses often fail to recognize delirium, resulting in many patients experiencing delirium at night and subsequently pulling out their tubes.”*

(P13)

Four participants described knowledge gaps regarding hypoactive delirium. Some were unfamiliar with the subtype, while others perceived quiet patients as presenting less immediate risk and therefore afforded them less attention. One nurse stated:

*“We feel that the risk coefficient for this type of patient (hypoactive delirium) is relatively low, and the risk of extubation is low, so our attention to them is not as great as for the hyperactive or completely manic type.”*

(P03)

Reliance on individual experience created variation across staff, particularly where formal education was infrequent. Seven participants identified insufficient or overly theoretical training and favoured practical, scenario-based learning that combined assessment, communication, patient and staff protection, and interprofessional response. One participant suggested:

*“I hope we can have some kind of practical combat drill… when you really encounter a delirious patient, how do you protect yourself, how do you protect the patient, how do you assist the doctor, how to handle it.”*

(P05)

A targeted post hoc comparison undertaken during revision did not identify a consistent capability- or motivation-related pattern distinguishing the three master’s-prepared nurses from bachelor’s-prepared nurses. All three master’s-prepared participants were also classified as senior nurses, with ICU experience ranging from 4 to 16 years, so educational preparation could not be disentangled from seniority, role, or accumulated clinical exposure. The study was therefore not considered suitable for degree-based subgroup inference.

### 3.4. Opportunity

#### 3.4.1. Environment and Systemic Constraints

Five participants described fragmented or single-room layouts as reducing visual access and making continuous observation difficult when one nurse was responsible for patients in different rooms. One nurse explained:

*“If I manage three patients, they might be distributed in three different rooms. If one of them is a delirious patient, the management difficulty is much greater because I cannot always stay by his side.”*

(P14)

Nine participants from both hospitals and all ICU types described sensory overstimulation, including alarms, continuous lighting, procedural activity, and disrupted day–night cues. These conditions were perceived to disturb sleep, increase anxiety, and complicate delirium prevention and management. One participant recalled:

*“The intensive care unit typically maintains continuous lighting for 24 h. At night, if critically ill patients require interventions, the lights cannot be turned off; when patients are stable, some lights may be dimmed while others remain on. This lack of distinction between day and night disrupts patients’ circadian rhythms, which can significantly contribute to the development of delirium. Additionally, the persistent noise from monitor alarms can increase patient anxiety and physiological stress. These environmental factors, combined with pharmacological agents, may precipitate or exacerbate delirium, thereby increasing the burden of patient management.”*

(P09)

Five participants described staff shortages, and 12 described high-acuity workload as constraining sustained observation and non-pharmacological care. Participants reported prioritising physiological instability and time-critical tasks, with delirium care becoming more reactive when several patients required simultaneous attention. One participant explained:

*“I’m usually handling three to four patients, and when it’s chaotic and noisy around me, I just don’t have much time for non-pharmacological interventions. To be honest, a lot of the time I have to prioritize the more critical patients and just ask the doctor to manage them with medication so I can free up my hands. It’s kind of unavoidable when the workload is really heavy, you know.”*

(P12)

Five participants explicitly described adequate staffing as a facilitator because it enabled closer observation, more frequent communication, and greater use of non-pharmacological strategies. One participant stated:

*“If staffing were more adequate, we could communicate with conscious patients more frequently and provide psychological care. That might reduce the occurrence of delirium and help improve its symptoms”*

(P04)

Six participants also described controlled lighting, reduced noise, and clearer day–night cues as facilitating sleep promotion and reorientation. One nurse explained:

*“We can turn off the bedside light, avoid direct light, and ask families to provide eye masks and earplugs to create a better rest environment. During the day, we tell patients that it is daytime and what time it is to help re-establish temporal orientation”*

(P04)

#### 3.4.2. Delirium Management Is a Team Sport

Seven participants described peer assistance as a reliable source of support during acute agitation, enabling nurses to share observation, restraint-related tasks, and competing workload. Eleven participants described supportive physician collaboration, whereas two reported delayed or “wait-and-see” responses. These were not mutually exclusive at the dataset level: some accounts described responsive collaboration in one context and delayed escalation in another. One participant describing delayed response stated:

*“Most of the time, when doctors know a patient has developed delirium, they adopt a wait-and-see attitude, thinking the patient will naturally wake up once the drug metabolizes.”*

(P09)

Ten participants described family engagement as a valuable non-pharmacological resource. Familiar voices and presence were perceived to reduce fear, support reorientation, and improve acceptance of treatment in ways that staff communication could not always reproduce. One nurse stated:

*“Many patients with delirium, especially the elderly, become anxious as soon as they’re admitted to the ICU—just because of the unfamiliar environment and not being able to see their family. Some even become aggressive out of fear. We can try over and over to explain why treatment is necessary and calm them down, but it barely helps. But the moment a family member comes in—even if they just sit by the bedside and talk about everyday things—the patient often settles down quickly. Their voice and presence provide the kind of comfort that feels most familiar and reassuring, and it immediately eases that fear of separation. Honestly, it works better than anything we can say.”*

(P08)

Four participants described restrictive visiting arrangements as limiting this relational resource. Although exceptions were sometimes arranged for selected patients, short and infrequent visits were perceived as insufficient for sustained reassurance. One participant explained:

*“The visitation policy is from 3:30 to 4:00 in the afternoon, and only one family member per patient is allowed per visit… Honestly, I don’t think it helps much with improving delirium in patients. The visiting time is just too short.”*

(P09)

### 3.5. Motivation

#### 3.5.1. Safety First, at Any Cost

Safety concerns were reported across both hospitals and all ICU types. Twelve participants described risks of unplanned device removal, nine described falls, and 11 described injury or violence toward staff when patients were severely agitated. These concerns were grounded in previous clinical incidents and shaped which responses were considered immediately feasible. One participant recalled:

*“One particularly strong patient, when exhibiting aggressive behaviour, used his head to strike our colleague’s head, knocking her unconscious right there.”*

(P06)

Safety-oriented motivation was accompanied by professional duty, described by four participants, and accountability for preventable adverse events, described by seven. Participants reported continuing to intervene despite physical effort, emotional discomfort, or limited treatment options because responsibility for patient safety could not be set aside. One participant stated:

*“The duty is there, the responsibility is there, it’s impossible to ignore.”*

(P02)

All 14 participants referred to physical or protective restraint as a response to agitation or immediate safety concerns, particularly risks of device removal, falls, or injury when verbal reassurance was ineffective. They presented restraint as a situational safety response rather than a preferred delirium intervention. The study did not evaluate its effectiveness, appropriateness, or adherence to local restraint protocols.

*“If clear cognitive change has developed into delirium, we first use restraint to protect ourselves and the patient, and we communicate with the doctor about analgesia or sedation. I do not like restraining patients, but sometimes we feel there is no alternative.”*

(P01)

#### 3.5.2. Weathering the Emotional Storm

Twelve participants described emotional burden arising from persistent agitation or aggression; three described helplessness, and five specifically discussed verbal abuse. Verbal abuse was especially difficult because nurses could recognise the behaviour as illness-related while still experiencing discomfort and a sense of being wronged. One nurse explained:

*“The most unacceptable is their verbal abuse towards medical staff. Although knowing the patient doesn’t mean it, hearing it still makes you feel uncomfortable and wronged.”*

(P09)

Patient age was mentioned inconsistently in accounts of aggression. One participant distinguished profanity from some older patients from physical aggression by younger men, but patient age was not systematically documented in episodes specifically involving verbal abuse, and nurses’ emotional responses were not compared by age group. The data therefore did not support an older-versus-younger comparison of the emotional effects of verbal abuse.

Ten participants described cognitive empathy as helping them reinterpret aggression as a manifestation of illness rather than intentional hostility. This reframing supported continued engagement and reduced personalisation of patients’ words and actions. One participant reflected:

*“Let flowers be flowers, let trees be trees, don’t force too much… able to understand the existence of this behaviour, and respond accordingly, won’t be affected by some of the patient’s behaviours or words.”*

(P05)

One participant described setting an explicit emotional boundary in care, while two described disengaging from work-related distress after handover. These strategies were framed as practical ways of recovering between shifts rather than as complete protection from cumulative emotional strain. One of the two participants who described off-duty disengagement commented:

*“If there is really no way to intervene, and they remain continuously restless, then the only way is to forget it after getting off work. Just handing over the shift, that is the best solution, just quickly get off work.”*

(P03)

#### Cross-Domain COM-B Interactions

Although the findings are presented by COM-B domain for analytic clarity, participants’ accounts frequently linked domains within the same bedside episode. P09 described reduced night staffing (opportunity) as making it difficult to maintain observation; when delirium developed suddenly, this constraint increased anxiety (motivation) and could narrow the immediate behavioural response toward restraint. P02 similarly described an increasing caseload as reducing the time available for relational care and producing a sense of helplessness, despite recognising the patient’s needs. Conversely, P12 described team leaders or senior nurses stepping in when younger nurses became nervous or frustrated, indicating that experiential capability and social opportunity could buffer negative motivational responses.

These cross-domain pathways show why capability alone did not determine behaviour. A nurse could recognise delirium yet still be unable to sustain non-pharmacological care when visibility, staffing, workload, or medical responsiveness constrained opportunity; under those conditions, safety-oriented motivation and emotional strain made rapid containment more immediately feasible. By contrast, peer, senior, physician, and family support expanded the range of feasible relational responses.

## 4. Discussion

This study identified 18 barriers and 15 facilitators across six themes within the COM-B domains. Its principal contribution is not simply to confirm that knowledge, workload, and organisational barriers exist, but to demonstrate how capability, opportunity, and motivation interacted in bedside decision-making. Three recurrent pathways were evident. First, assessment uncertainty around hypoactive delirium and tool interpretation became more consequential when workload or fragmented visibility limited the opportunity for sustained interaction. Second, staffing and workload constraints could amplify anxiety, helplessness, and safety-first motivation, narrowing the range of immediately feasible responses toward restraint or medication. Third, social opportunity through senior, peer, physician, and family support could buffer emotional burden and help nurses sustain relational or non-pharmacological care. The COM-B domains therefore functioned as interdependent determinants rather than parallel descriptive categories [12,14].

Safety-first reasoning was central to motivation. Participants described protective restraint and medication as situational responses when device removal, falls, or violence appeared imminent, particularly when reassurance was ineffective or observation could not be sustained. These accounts should not be interpreted as evidence that restraint is an effective or recommended delirium intervention. Rather, they illustrate how perceived responsibility and limited alternatives can narrow the actions nurses regard as feasible. Importantly, participants framed accountability mainly in terms of preventable adverse events and nursing responsibility; they did not identify a specific Chinese law, legal provision, or institutional restraint policy as the mechanism driving these concerns. The present data therefore support an interpretation of perceived clinical/professional accountability, not a legal-policy causal claim. This interpretation is consistent with research describing restraint decisions as a balance among patient protection, staff safety, and operational demands [21,22].

Physical opportunity placed an upper limit on what nurses could deliver. Fragmented room layouts reduced visibility, sensory stimulation disrupted sleep and orientation, and staffing shortages restricted sustained communication and observation. Conversely, participants explicitly described adequate staffing and controlled lighting and noise as enabling closer surveillance and non-pharmacological care. These findings align with studies showing that delirium practices are difficult to normalise when staffing, workflow, and the ICU environment do not support them [23,24,25,26,27]. Environmental and workforce recommendations arising from this study should nevertheless be treated as practice directions for evaluation, not as interventions whose effectiveness was tested here.

Social opportunity was equally influential. Peer support redistributed workload during episodes of agitation, while physician responsiveness determined whether nurses could escalate assessment and treatment promptly. Family engagement was particularly salient: familiar voices were perceived to reduce fear and support reorientation, whereas restrictive visiting policies removed an important relational resource. The finding is contextually important because YC’s previous ICU visitation research could have increased sensitivity to family-related accounts; these interpretations were therefore explicitly discussed with YG and examined against the complete dataset. Family effects in these interviews were expressed primarily through familiarity, reassurance, presence, and improved cooperation. No separate theme corresponding to filial piety emerged, so the data should not be extended to claim a specific filial-piety mechanism. The convergence of family-related accounts across both hospitals and multiple ICU types nevertheless supported family presence as a substantive social-opportunity finding.

Capability depended on more than factual knowledge. Hyperactive delirium was readily recognised, but hypoactive presentations and assessment of older adults required comparison with baseline cognition, sustained interaction, and confidence in interpreting responses. Inconsistent use of CAM-ICU reflected both practical limitations and uncertainty about applicability, rather than simple rejection of structured screening. Similar difficulties have been reported in other ICU settings [13,28,29,30,31,32]. Training should therefore address subtype recognition, communication barriers, and the integration of structured tools with clinical context.

Experience facilitated pattern recognition, anticipation of risk, and emotional composure; however, reliance on informal expertise also produced variability between senior and novice nurses. Participants consequently favoured scenario-based learning, mentoring, and accessible decision support. Educational interventions can improve knowledge and confidence [33], but the present findings suggest that gains are unlikely to persist without opportunities to practise, feedback from experienced colleagues, and organisational conditions that permit nurses to use what they have learned [12,14].

Emotional labour shaped motivation to sustain person-centred care. Participants described frustration, helplessness, verbal abuse, and violence, while cognitive empathy, emotional boundaries, and off-duty disengagement helped them continue working. These strategies were individually useful but did not replace organisational support. Repeated exposure to aggression without debriefing or visible support may make containment-focused responses more likely on subsequent shifts [34,35]. Psychological safety, brief post-incident review, and clear escalation arrangements may therefore warrant evaluation alongside technical education.

To translate these determinants into intervention design without implying tested effectiveness, key findings were mapped to Behaviour Change Wheel intervention functions (Table 3) [15]. The most consistently relevant functions were Training, Environmental restructuring, Enablement, and Modelling. Education was also relevant for delirium subtype knowledge and structured assessment, but the interaction findings indicate that information alone is unlikely to change behaviour when opportunity remains constrained or when nurses lack timely practical alternatives. The mapping is therefore hypothesis-generating and identifies candidate strategies for prospective development and evaluation rather than interventions demonstrated to be effective in this study.

Taken together, the findings indicate that delirium management among ICU nurses is produced by dynamic interaction among capability, opportunity, and motivation rather than by knowledge deficits alone. For the participating hospitals, the data prioritise four Behaviour Change Wheel functions for prospective evaluation: Training to strengthen practical recognition and de-escalation skills; Environmental restructuring to improve visibility, sleep-promoting conditions, family access, and escalation pathways; Enablement to make timely assistance and feasible alternatives available; and Modelling through senior mentoring and team-based practice. Education remains necessary but is unlikely to be sufficient in isolation. Because this qualitative study did not test these strategies, they should be understood as empirically grounded directions rather than demonstrated solutions.

## 5. Limitations

Several limitations should be considered. First, the small purposive sample was drawn from two urban tertiary hospitals, with 11 participants from Site 1 and only three from Site 2. Data adequacy was assessed across the combined sample, not separately by site, so the study does not support site-specific saturation or formal between-hospital comparison; transferability should be judged contextually rather than statistically. Second, the sample comprised nurses only, and self-reported accounts were not triangulated with observation, policy documents, or perspectives from physicians, patients, or families. Third, recruitment was facilitated by a workplace colleague at Site 1, and a senior author held a nursing administrative role at that hospital. Although the interviewer had no prior working relationship with participants, interviews were private, and individual participation decisions were not disclosed to nursing managers, selection effects, perceived professional pressure, and socially desirable responding cannot be excluded. Fourth, the revision-stage independent subset coding strengthened verification of participant-level factor assignment but used the already fixed 33-factor codebook; it was not a de novo second thematic analysis and therefore cannot completely remove the influence of the original analytic structure. Fifth, all analysis was conducted in Mandarin and selected quotations were translated only for reporting. Conceptual equivalence and affective nuance were checked against the Mandarin source by YC and YG, but no independent back-translation was undertaken. Sixth, field notes, transcript return, and participant checking were not used, and the number of eligible nurses approached or declining participation was not formally recorded. Credibility was instead supported through cross-case comparison, divergent evidence, full-dataset audit, and independent subset coding, but participant feedback could have provided an additional interpretive perspective. Seventh, patient age was not consistently documented in verbal-abuse narratives, precluding a reliable comparison of nurses’ emotional responses to abuse by older versus younger patients. Finally, accounts of physical restraint and accountability were not triangulated with direct observation, local protocol review, or legal/policy documents; they should therefore be interpreted as perceived clinical responsibility and situational practice rather than evidence of policy compliance, legal causation, or endorsement of restraint.

## 6. Conclusions

Among 14 ICU nurses from two tertiary hospitals in Chongqing, delirium management was shaped by interacting factors related to capability, opportunity, and motivation. Experience and conversational assessment supported recognition, while hypoactive presentations, workload, fragmented visibility, restricted family access, safety risks, and emotional burden constrained sustained person-centred care. Cross-domain accounts showed that opportunity constraints could amplify safety-oriented and emotional motivation, while social support could preserve relational care. The findings suggest Training, Environmental restructuring, Enablement, and Modelling as priority Behaviour Change Wheel functions for prospective intervention development, while reinforcing that education alone is unlikely to be sufficient. These practice directions require prospective evaluation and should not be interpreted as already demonstrated solutions.

## Figures and Tables

**Table 1:** Participant characteristics.

Participant	Site/ICU	Age/Sex	Education	Professional Level/Role/ICU Years	Prior Delirium Training/Standardised Protocol
P01	Site 1/General ICU	30/F	Master	Senior nurse/Bedside/4	Yes/No
P02	Site 1/General ICU	28/F	Bachelor	Nurse/Bedside/5	Yes/No
P03	Site 1/General ICU	28/M	Bachelor	Nurse/Bedside/5	Yes/No
P04	Site 1/EICU	33/F	Bachelor	Senior nurse/Educator/10	Yes/No
P05	Site 1/General ICU	35/F	Bachelor	Senior nurse/Bedside/10	Yes/No
P06	Site 1/General ICU	30/F	Bachelor	Nurse/Bedside/6	Yes/Yes
P07	Site 1/EICU	38/F	Bachelor	Senior nurse/Team leader/10	Yes/Yes
P08	Site 1/EICU	36/F	Master	Senior nurse/Team leader/10	Yes/No
P09	Site 1/RICU	43/F	Master	Senior nurse/Team leader/16	No/No
P10	Site 1/RICU	32/F	Bachelor	Senior nurse/Bedside/8	Yes/No
P11	Site 1/RICU	28/M	Bachelor	Nurse/Bedside/6	Yes/No
P12	Site 2/General ICU	40/F	Bachelor	Senior nurse/Team leader/18	Yes/Yes
P13	Site 2/General ICU	33/M	Bachelor	Senior nurse/Bedside/11	Yes/Yes
P14	Site 2/General ICU	25/M	Bachelor	Junior nurse/Bedside/2	No/No

Abbreviations: EICU, emergency intensive care unit; RICU, respiratory intensive care unit. Site 1, The Second Affiliated Hospital of Chongqing Medical University; Site 2, Army Medical Center (Daping Hospital).

**Table 2:** COM-B domains, themes, barriers, and facilitators.

COM-B Domain	Theme	Lowest-Level Barriers and Facilitators
Capability	1. Navigating the complexities of assessment	**Facilitators:** Hyperactive observation; conversational assessment **Barriers:** Hypoactive delirium; older-adult comprehension; CAM-ICU difficulties
	2. Perception of clinical experience and training	**Facilitators:** Clinical experience; senior intuition; subtle cues **Barriers:** Novice inexperience; hypoactive-delirium knowledge gaps; insufficient training
Opportunity	3. Environment and systemic constraints	**Facilitators:** Adequate staffing; controlled environment **Barriers:** Single-room layouts; sensory overstimulation; staff shortages; high workload
	4. Delirium management is a team sport	**Facilitators:** Family engagement; peer support; physician collaboration **Barriers:** Restrictive visiting policies; delayed medical response
Motivation	5. Safety first, at any cost	**Facilitators:** Professional duty; accountability **Barriers:** Device-removal risk; falls; staff violence
	6. Weathering the emotional storm	**Facilitators:** Cognitive empathy; emotional boundaries; off-duty disengagement **Barriers:** Emotional toll; helplessness; verbal abuse

**Table 3:** Mapping key COM-B determinants to Behaviour Change Wheel intervention functions and candidate ICU practice strategies.

COM-B Determinant (s)	Study Finding	BCW Intervention Function (s)	Candidate Strategy for Evaluation
Capability: hypoactive-delirium knowledge gaps; CAM-ICU difficulties; insufficient training	Recognition and tool use were limited by subtype uncertainty, patient comprehension, and requests for more practical preparation.	Education; Training	Brief subtype-focused education plus repeated CAM-ICU practice, communication adaptations, and scenario simulation.
Capability + social opportunity: novice inexperience; clinical experience; senior intuition	Experience improved pattern recognition and composure; senior nurses provided practical support to less-experienced staff.	Training; Modelling; Enablement	Mentored case review, bedside coaching, simulation with senior role-modelling, and accessible decision support.
Physical opportunity: staff shortages; high workload; fragmented room layouts	Competing demands and reduced visibility limited sustained observation and non-pharmacological care.	Environmental restructuring; Enablement	Review staffing/assignment patterns, visibility supports, task redistribution, and rapid backup for high-risk patients.
Physical opportunity: sensory overstimulation; controlled environment	Light, alarms, and night-time activity disrupted sleep and orientation, while feasible environmental control was perceived as helpful.	Environmental restructuring	Sleep-promoting light/noise routines, day–night orientation cues, and practical environmental modifications.
Social opportunity: restrictive visiting policies; family engagement	Family presence and familiar communication could calm and reorient patients, but restricted access limited this resource.	Environmental restructuring; Enablement	Criteria-based flexible family presence and structured guidance for family-supported reorientation when clinically appropriate.
Social opportunity: physician collaboration; delayed medical response	Responsive medical input expanded nursing options, whereas wait-and-see responses constrained timely escalation.	Environmental restructuring; Enablement	Explicit escalation pathways, shared delirium response expectations, and rapid interprofessional review triggers.
Motivation + opportunity: device-removal/fall risk; professional duty; accountability	Perceived responsibility for preventable harm could narrow feasible responses toward immediate containment when alternatives were not readily available.	Training; Enablement; Modelling	Practice de-escalation and least-restrictive alternatives, provide rapid assistance, and model risk-balanced decision-making.
Motivation: verbal abuse; staff violence; emotional toll; helplessness	Aggression and emotional burden threatened sustained person-centred engagement; empathy and coping strategies offered partial protection.	Training; Enablement	De-escalation and staff-safety training, brief post-incident review, psychological support, and clear escalation/debriefing options.

## Data Availability

The data that support the findings of this study are not publicly available due to privacy and ethical restrictions. De-identified data may be available from the corresponding author upon reasonable request and subject to approval by the relevant ethics committee.

## Supplementary Materials

The following supporting information can be downloaded at https://www.mdpi.com/article/10.3390/healthcare14162646/s1, Supplementary File S1, COREQ Checklist; Supplementary File S2, Interview Guide: “Barriers and Facilitators to Delirium Management among ICU Nurses”; Supplementary File S3, Revision-stage Fixed-codebook Concordance Audit; Supplementary File S4, Revision-stage independent subset coding concordance and consensus resolution.
