# Supplementary material for: Barriers and Facilitators to Delirium Management Among ICU Nurses in Two Tertiary Hospitals in Chongqing, China: A Qualitative Study Using the COM-B Framework
Source: Healthcare (Basel). 2026 Aug 20;14(16):2646. doi: 10.3390/healthcare14162646 (PMC13512898; doi:10.3390/healthcare14162646)
Supplement: Supplementary file 1 [file healthcare-14-02646-s001.zip › Supplementary File S1.pdf]

Supplementary File S1: COREQ Checklist

	COREQ Item	Answer
Domain 1: Research Team and Reflexivity		
1	Interviewer/facilitator- Which author/s conducted the interview or focus group?	YC conducted all 14 interviews. YG did not conduct interviews. During the revision-stage verification, YG independently applied the fixed 33-factor COM-B codebook to five complete Mandarin transcripts (P02, P04, P09, P12, and P14) before viewing YC's transcript-level assignments.
2	Credentials-What were the researcher's credentials? E.g., PhD, MD	YC- MD<br>YG- PhD<br>RY-RN,PhD<br>XL- RN<br>XyL- MD<br>XW-MD<br>XG-Professor<br>XS- Associate Professor
3	Occupation-What was their occupation at the time of the study?	Clinical nursing, nursing education, research, medical practice.
4	Gender-Was the researcher male or female?	YC- Male<br>YG-Female<br>RY-Female<br>XL-Female<br>XyL-Female<br>XW-Female<br>XG-Female<br>XS-Female
5	Experience and training-What experience or training did the researcher have?	YC had prior experience in qualitative data collection and analysis through participation in an earlier ICU visitation study, but was not a delirium specialist or an expert qualitative methodologist. YG had previous research and analytic experience.

6	Relationship established-Was a relationship established prior to study commencement?	YC and YG had no prior working relationship with participants. RY was an ICU colleague of some potential participants at Site 1 and facilitated initial contact but did not conduct interviews, transcribe data, or undertake coding. XG held a senior nursing administrative role at Site 1 but was not the participants' immediate clinical supervisor and was not informed of individual decisions to participate or decline. XS facilitated access to Site 2 and had no employment or supervisory relationship with participants there.
7	Participant knowledge of the interviewer-What did the participants know about the researcher? e.g., personal goals, reasons for doing the research.	Participants were informed that the study aimed to understand ICU nurses' experiences of delirium assessment and management and identify potential improvements in clinical practice. Individual decisions to participate or decline were not disclosed to nursing managers.
8	Interviewer characteristics-What characteristics were reported about the interviewer/facilitator? e.g. Bias, assumptions, reasons and interests in the research topic.	YC had a professional interest in ICU delirium and prior involvement in an ICU visitation study, which was treated as a potential sensitising influence on family-related accounts. Recruitment relationships were also considered reflexively because RY was a colleague of some Site 1 participants and XG held a senior administrative role. RY did not interview or code data; interviews were conducted privately by YC, who had no prior working relationship with participants, and individual participation decisions were not disclosed to nursing managers. These safeguards reduced but could not eliminate selection or social-desirability effects. Family-related and other interpretations were discussed with YG and checked against the complete dataset, including limiting or contrasting accounts.

Domain 2: Study Design		
9	Methodological orientation and Theory-What methodological orientation was stated to underpin the study? e.g., grounded theory, discourse analysis, ethnography, phenomenology, content analysis	A qualitative descriptive exploratory (QDE) methodology was used. The study was guided by the Capability, Opportunity and Motivation model of Behaviour (COM-B). After the thematic structure had been finalised and verified, selected determinants were mapped to Behaviour Change Wheel (BCW) intervention functions as an interpretive step to generate candidate practice strategies; this mapping did not contribute to theme generation or constitute intervention testing.
10	Sampling-How were participants selected? e.g., purposive, convenience, consecutive, snowball	Purposive sampling was used to recruit nurses with diverse clinical experience, educational qualifications, and ICU backgrounds.
11	Method of approach-How were participants approached? e.g., face-to-face, telephone, mail, email	At Site 1, RY circulated study information and facilitated initial contact; at Site 2, XS facilitated access. Willing participants then completed private, face-to-face interviews with YC. The number approached or declining was not formally recorded.
12	Sample size-How many participants were in the study?	14 participants.
13	Non-participation-How many people refused to participate or dropped out? Reasons?	The number declining participation was not formally recorded. No participant withdrew after providing written informed consent.
14	Setting of data collection-Where was the data collected? e.g., home, clinic, workplace	Interviews were conducted face-to-face in a private room at the participants' workplace during participants' voluntary lunch breaks or after their shifts. No interviews were interrupted.
15	Presence of non-participants-Was anyone else present besides the participants and researchers?	No managers, colleagues, or other non-participants were present.
16	Description of sample-What are the important characteristics of the sample? e.g., demographic data, date	Registered ICU nurses, males and females, participants with bachelor's and master's degrees, and participants with varied ICU work experience across comprehensive, emergency, and respiratory ICU settings.

17	Interview guide-Were questions, prompts, guides provided by the authors? Was it pilot tested?	Yes. A semi-structured interview guide based on the COM-B model was pilot-tested with three nurses of similar clinical backgrounds. The guide was revised following the pilot interviews. Pilot interview data were not coded and were not included in the formal analysis.
18	Repeat interviews-Were repeat interviews carried out? If yes, how many?	No
19	Audio/visual recording-Did the research use audio or visual recording to collect the data?	Yes. Interviews were audio-recorded.
20	Field notes-Were field notes made during and/or after the interview or focus group?	No field notes were made.
21	Duration-What was the duration of the interviews or focus group?	Interviews lasted between 24 and 37 minutes.
22	Data saturation-Was data saturation discussed?	Yes. Data collection and preliminary analysis proceeded concurrently. Data adequacy was assessed across the combined purposive sample rather than separately within each hospital. By interview 12, no substantively new codes or themes were identified, and two additional interviews were conducted to check that the developing thematic structure remained adequate, resulting in 14 interviews. A formal saturation log was not maintained. Because Site 2 contributed three participants, the study does not claim site-specific saturation or support formal between-site thematic comparison.
23	Transcripts returned-Were transcripts returned to participants for comment and/or correction?	No. Transcripts were not returned to participants for comment or correction.
Domain 3: Analysis and Findings		

24	Number of data coders-How many data coders coded the data?	YC completed the initial coding of all 14 Mandarin transcripts. During revision, YG independently applied the fixed 33-factor codebook to five complete transcripts (P02, P04, P09, P12, and P14) selected for variation across site, ICU type, experience, and education. YG completed first-pass coding before viewing YC's transcript-level assignments. At the participant-by-factor level, 137 of 165 coding decisions were initially concordant (83.0%; descriptive Cohen's kappa = 0.656), leaving 28 disagreements. YC and YG resolved each disagreement by returning to the original Mandarin passage and surrounding context and applying the prespecified operational definition and inclusion/exclusion boundary; neither coder's initial assignment was treated as the default. Ten disagreements retained the YC/full-audit interpretation and 18 retained the YG interpretation. The reconciliation did not alter the six-theme, 18-barrier/15-facilitator structure (Supplementary File S2).

25	Description of the coding tree -Did authors provide a description of the coding tree?	Yes. Table 2 of the manuscript shows the relationship between the three COM-B domains, six themes, and 33 lowest-level factors (18 barriers and 15 facilitators). Supplementary File S1 provides the revision-stage fixed-codebook audit across all 14 Mandarin transcripts, including operational definitions, inclusion/exclusion boundaries, supporting coverage, and contrasting/boundary evidence. Supplementary File S2 documents YG's independent five-transcript subset coding, YC-YG comparison, disagreement resolution, and final consensus. These revision-stage documents audit the fixed analytic structure and do not replace or purport to reproduce the original codebook.
26	Derivation of themes-Were themes identified in advance or derived from the data?	The three COM-B domains were specified deductively as organising categories. The six themes and their lowest-level barriers and facilitators were developed from the interview data within those domains. The revision-stage fixed-codebook audit and independent subset recoding were verification procedures and did not re-generate the thematic structure.
27	Software-What software, if applicable, was used to manage the data?	YC used NVivo 12 to code the Mandarin transcripts.

28	Participant checking-Did participants provide feedback on the findings?	No. Transcripts were not returned and participant checking of the findings was not undertaken. Credibility was instead supported through repeated transcript reading, cross-case comparison, examination of divergent/contrasting accounts, direct linkage to Mandarin transcript evidence, a full-dataset fixed-codebook audit, and revision-stage independent subset coding. The absence of participant checking is reported as a limitation.
29	Quotations presented-Were participant quotations presented to illustrate the themes/findings? Was each quotation identified? e.g., participant number	Yes, to both.
30	Data and findings consistent-Was there consistency between the data presented and the findings?	Yes. Consistency between the data and findings was checked through direct linkage of all 33 fixed factors to the 14 Mandarin transcripts (Supplementary File S1), preservation of boundary/contrasting evidence, and YG's independent subset coding with documented YC-YG consensus resolution (Supplementary File S2).
31	Clarity of major themes-Were major themes clearly presented in the findings?	Yes
32	Clarity of minor themes-Is there a description of diverse cases or discussion of minor themes?	Yes
