# Supplementary material for: Barriers and Facilitators to Delirium Management Among ICU Nurses in Two Tertiary Hospitals in Chongqing, China: A Qualitative Study Using the COM-B Framework
Source: Healthcare (Basel). 2026 Aug 20;14(16):2646. doi: 10.3390/healthcare14162646 (PMC13512898; doi:10.3390/healthcare14162646)
Supplement: Supplementary file 1 [file healthcare-14-02646-s001.zip › Supplementary File S2.pdf]

Supplementary File S2: Interview Guide: "Barriers and Facilitators to Delirium Management among ICU Nurses"

(Translated from Chinese)

1. Participant background information

Before the interview, participants were asked to complete a brief demographic form, including age, gender, highest educational level, professional title, current role, years of ICU experience, ICU type, previous delirium-related training, and whether a standardised delirium management protocol was available in their unit.

2. Opening and warm-up questions

Question 1. How long have you worked in the ICU? What are the characteristics of your current shift or role (e.g. number of patients under your care, teaching responsibilities, or whether you often care for awake patients)?

> ***Probe prompts:*** What is the biggest difference between day and night shifts in delirium care?

Question 2. Can you describe the most recent delirious patient you cared for in the ICU? How did you feel about that experience overall?

> ***Probe prompts:*** How did you first realise that something was wrong? What was the first action you took, and why (e.g. reassurance, restraint, or contacting a physician)? Looking back, what was the greatest source of pressure (e.g. aggressive behaviour, unplanned device removal, or family complaints)?

3. Main interview questions

Capability

Question 3. How would you describe your ability to care for patients with delirium? What do you find most difficult?

> ***Probe prompts:*** In recognising and assessing delirium, where do you feel most uncertain or find the greatest difficulty? When using delirium screening tools or scales, what specific difficulties do you encounter—for example, not knowing how to use them, not having enough time, or not trusting the result after completing the assessment? When managing different delirium subtypes (hyperactive, hypoactive, or mixed), which procedures or decisions feel most challenging?

Question 4. How confident are you in making judgments and managing patients with delirium? How do you deal with uncertainty?

> ***Probe prompts:*** When a patient's symptoms are complex or change rapidly, how do you usually make decisions? When you are uncertain or worried about making a mistake, how do you respond?

Question 5. From the perspective of physical and mental energy, how does delirium care affect you?

> ***Probe prompts:*** Compared with caring for other patients, how is the energy expenditure different when caring for a patient with delirium? Do prolonged observation, repeated

reassurance, and prevention of device removal make you feel especially fatigued? What do you do to relieve this physical and mental fatigue?

Question 6. What kinds of training or tools would help you most in delirium care?

Probe prompts: Which areas of training do you need most—for example, delirium knowledge, use of assessment tools, management strategies for different delirium subtypes, or medication-related knowledge? What training formats do you prefer—for example, theoretical teaching plus case discussion, scenario simulation or role-play, online microlearning, or bedside coaching? What aspects of the current training do you think should be improved?

Opportunity

Question 7. In teamwork and collaboration, what factors influence your delirium care?

Probe prompts: How do physicians' attitudes and level of collaboration in delirium management make care easier or more difficult for you? When working with other nurses or the multidisciplinary team, what aspects of collaboration work well, and what aspects are difficult? When family members are involved, what do you find particularly helpful? Are there any aspects of family involvement that increase pressure or make delirium care more difficult?

Question 8. How do the ICU physical environment and available resources (e.g. equipment, bed layout, monitoring systems, staffing, time arrangements) affect your management of patients with delirium?

Probe prompts: Does staffing affect whether you have enough time to assess and intervene for delirium? Are monitoring equipment, restraint devices, and safety facilities sufficient and practical to use? Do environmental factors such as lighting, noise, and visiting arrangements aggravate or alleviate delirium? Do these factors make your work more difficult or easier?

Question 9. What support would you like your ward or hospital to provide?

Probe prompts: In which areas would you like more staffing or equipment? Which ward policies or processes—for example, standardised delirium screening, prevention, and management pathways—need improvement? What changes would you most like to see in multidisciplinary teamwork?

Motivation

Question 10. What emotional experiences do you usually have when caring for patients with delirium?

Probe prompts: In which situations do you feel particularly nervous or afraid? Have you ever felt extremely frustrated or wanted to avoid the situation? How do you regulate or manage your emotions?

Question 11. How important do you think delirium care is in your daily work?

Probe prompts: How strongly do you think delirium care is related to patient outcomes and nursing quality? When you have many tasks and are very busy, how do you prioritise

delirium care relative to other tasks? Are there any principles or beliefs that influence how much effort you devote to delirium care?

Question 12. What would increase your motivation to provide delirium care?

Probe prompts: Does recognition or feedback from supervisors or the team affect you? How would you prefer to be recognised? Has your ward provided psychological support, stress-reduction activities, or peer sharing, and have these been helpful? What institutional or cultural changes do you think would make staff more willing to provide proactive delirium care?

Question 13. Compared with when you first started working in the ICU, how have your feelings and attitudes towards delirium care changed?

Probe prompts: Which aspects have improved with experience, which have become more challenging, and why do you think these changes occurred?

4. Closing question

Question 14. Is there anything else you would like to add that we have not discussed?
