# Supplementary material for: Barriers and Facilitators to Delirium Management Among ICU Nurses in Two Tertiary Hospitals in Chongqing, China: A Qualitative Study Using the COM-B Framework
Source: Healthcare (Basel). 2026 Aug 20;14(16):2646. doi: 10.3390/healthcare14162646 (PMC13512898; doi:10.3390/healthcare14162646)
Supplement: Supplementary file 1 [file healthcare-14-02646-s001.zip › Supplementary File S3.pdf]

Supplementary File S3. Revision-stage Fixed-codebook Concordance Audit

COM-B barriers and facilitators to delirium management among ICU nurses

1. Purpose and scope

This document audits the fixed analytic structure reported in the revised manuscript: three COM-B domains, six themes, and 33 lowest-level factors (18 barriers and 15 facilitators). The purpose is to demonstrate transcript-to-factor concordance, clarify operational boundaries between adjacent factors, identify contrasting or boundary evidence, and document whether the existing structure remains defensible when checked against the 14 anonymised Mandarin interview transcripts (P01-P14).

The audit is deliberately fixed-codebook at the factor level. It does not present the 33 factors as newly generated during revision and does not replace the original analytic process. Each factor is checked against the Mandarin transcripts using its predefined meaning, with attention to direct supporting accounts and potentially competing interpretations. Representative excerpts are lightly trimmed for display; ellipses indicate omitted words or sentences, while the substantive wording is otherwise retained.

This full-dataset audit is not itself an inter-coder reliability exercise. Independent subset coding and the subsequent YC–YG comparison/disagreement-resolution procedure are documented in Supplementary File S2. Final consensus assignments from the five independently recoded transcripts (P02, P04, P09, P12, and P14) have been incorporated into the transcript coverage reported here.

2. Audit descriptors

Descriptor	Meaning in this audit
Strongly supported	Repeated direct support in seven or more participants. This is an audit descriptor only, not a quantitative validity threshold.
Supported	Direct support in three to six participants, with a distinct operational meaning.
Supported with limited recurrence	Direct support in one or two participants. Retained only where the meaning is analytically distinct and not better represented by an adjacent factor.
Boundary/contrasting evidence	Accounts that qualify, limit, or contrast with the factor; such evidence is used to refine claims rather than treated as a reason to suppress the factor.

3. Overall concordance summary

All 33 fixed factors retained identifiable transcript support after integration of the YC–YG subset consensus. Sixteen factors were classified as strongly supported, thirteen as supported, and four as supported with limited recurrence. The four lower-recurrence factors were Older-adult comprehension (P02, P04), Delayed medical response (P07, P09), Emotional boundaries (P04), and Off-duty disengagement (P03, P11). Their retention is based on distinct

operational meaning and direct transcript evidence, not frequency alone. No factor was re-labelled as a new barrier or facilitator during this fixed-codebook audit.

Capability: Navigating the complexities of assessment

Factor / type	Operational definition	Inclusion / exclusion boundary	Transcript coverage	Representative transcript evidence	Concordance decision / boundary note

Factor / type	Operational definition	Inclusion / exclusion boundary	Transcript coverage	Representative transcript evidence	Concordance decision / boundary note
**1. Hyperactive observation** *Facilitator*	Recognition of delirium through overt agitation, disorganised speech, unsafe behaviour, or other readily observable hyperactive features.	Include accounts in which visible agitation or behavioural change makes delirium comparatively easy to notice. Exclude formal tool use and subtle/hypoactive presentations.	P01, P02, P03, P04, P06, P07, P08, P09, P10, P11, P12, P13, P14 n=13; both sites; EICU/General ICU/RICU	P02: 白天我们更关注躁动型（亢奋型）谵妄患者；相对而言，沉默型（低活动型）谵妄可能更少被注意到，也不太容易识别。 *During the day we pay more attention to agitated (hyperactive) delirium; comparatively, quiet (hypoactive) delirium may receive less attention and is harder to recognise.*	Strongly supported; retained. Contrasting accounts that quiet/hypoactive delirium was easier to miss strengthened the boundary between overt observation and hypoactive-recognition difficulty.

Factor / type	Operational definition	Inclusion / exclusion boundary	Transcript coverage	Representative transcript evidence	Concordance decision / boundary note
**2. Conversational assessment**<br>*Facilitator*	Use of simple orientation or conversational questions as a bedside supplement for identifying cognitive or attentional change.	Include active conversation used to judge orientation, coherence, or change from baseline. Exclude CAM-ICU-only assessment and general emotional reassurance without an assessment purpose.	P02, P04, P07, P10, P11, P13 n=6; both sites; EICU/General ICU/RICU	P13: 谵妄患者的行为挺多的。首先是眼神，跟正常人不一样。碰到这种情况，我会主动跟他聊天，不管有用没用，比如问哪里人、多大年纪、在哪里住院，多问几个问题。谵妄患者容易答错。<br>*Patients with delirium show many behaviours. The first clue may be their gaze, which looks different. In this situation I actively talk with them—for example asking where they are from, their age, and where they are hospitalised. Patients with delirium are likely to answer incorrectly.*	Supported; retained. Participants described conversation as a supplement rather than a universal replacement for structured assessment.

Factor / type	Operational definition	Inclusion / exclusion boundary	Transcript coverage	Representative transcript evidence	Concordance decision / boundary note
**3. Hypoactive delirium** *Barrier*	Difficulty detecting quiet, less agitated, or hypoactive delirium because behavioural signals are less conspicuous.	Include accounts of quiet/hypoactive delirium being overlooked or requiring deliberate interaction. Exclude explicit lack of knowledge of the subtype, which is coded separately.	P02, P03, P04, P07, P09, P10, P11, P13 n=8; both sites; EICU/General ICU/RICU	P13: 其实谵妄有躁狂型的，非常躁狂，也有安静型的。安静型不容易发觉，所以要主动聊天，判断他是不是答错问题。 *Delirium can be very agitated, but it can also be quiet. The quiet type is difficult to notice, so we need to initiate conversation and judge whether the patient is answering incorrectly.*	Strongly supported; retained. Some participants recognised hypoactive delirium and deliberately assessed it; the barrier therefore concerns detectability, not universal ignorance.

Factor / type	Operational definition	Inclusion / exclusion boundary	Transcript coverage	Representative transcript evidence	Concordance decision / boundary note
**4. Older-adult comprehension** *Barrier*	Uncertainty in distinguishing delirium-related responses from limited comprehension, baseline cognition, or communication difficulties in older adults.	Include accounts specifically linking older age/comprehension to uncertainty in interpretation. Exclude general CAM-ICU inconvenience without an age/comprehension component.	P02, P04 n=2; Site 1; EICU/General ICU	P04: 尤其一些老年患者，知识和理解能力本身有局限。很多时候会出现“所问非所答”，你也分不清他是本来就不知道，还是因为谵妄导致的答非所问、胡言乱语，所以不太好评估。 *Some older patients already have limitations in knowledge and comprehension. When their answers do not match the questions, it can be difficult to tell whether they simply do not know or whether delirium is causing incoherent responses, which makes assessment difficult.*	Supported with limited recurrence; retained. This factor was kept distinct from hypoactive delirium because the reported uncertainty concerned baseline understanding and response interpretation.

Factor / type	Operational definition	Inclusion / exclusion boundary	Transcript coverage	Representative transcript evidence	Concordance decision / boundary note
**5. CAM-ICU difficulties** *Barrier*	Practical or interpretive difficulty using CAM-ICU consistently in routine ICU care.	Include difficulty remembering items, obtaining usable responses, interpreting responses, or routinely applying the tool. Exclude complete absence of a delirium tool and general knowledge gaps unrelated to tool use.	P01, P02, P04, P05, P09, P11, P12 n=7; both sites; EICU/General ICU/RICU	P02: 我们科室用的是 CAM-ICU。我觉得实用性不是很强。前面两项相对还好观察，但后面要问问题时，很多患者不一定能理解你的问题。尤其七八十岁的老年患者，理解问题都很困难，更难保证能正确回答，所以评估更难。 *Our unit uses CAM-ICU. I do not find it very practical. The first items are easier to observe, but later questions can be difficult because many patients may not understand them, especially patients in their seventies or eighties, making assessment more difficult.*	Strongly supported; retained. Accounts varied: some participants used CAM-ICU routinely or regarded it as workable, indicating difficulty was not universal and should not be framed as rejection of structured screening. Retained; revised from “often refrain” to

Capability: Perception of clinical experience and training

Factor / type	Operational definition	Inclusion / exclusion boundary	Transcript coverage	Representative transcript evidence	Concordance decision / boundary note
**6. Clinical experience** *Facilitator*	Accumulated clinical exposure that improves pattern recognition, anticipation of delirium, and confidence in bedside judgement.	Include explicit comparisons between earlier and later practice or statements that seeing more cases improved recognition. Exclude rank-based senior support provided to others.	P02, P04, P07, P09, P11, P12, P13 n=7; both sites; EICU/General ICU/RICU	P11: 见的病例多了、接触的病情多了，心里就有了大致的判断标准，基本能准确判定患者是否发生谵妄了。 *After seeing more cases and a wider range of conditions, I developed an approximate internal standard and can generally judge whether delirium has occurred.*	Strongly supported; retained. Kept distinct from senior intuition: this factor concerns the individual's accumulated experience rather than immediate support from a senior colleague.

Factor / type	Operational definition	Inclusion / exclusion boundary	Transcript coverage	Representative transcript evidence	Concordance decision / boundary note
**7. Senior intuition** *Facilitator*	Rapid judgement and practical guidance provided by senior nurses or team leaders during difficult delirium episodes.	Include accounts in which senior staff step in, guide less experienced nurses, or use experience to stabilise management. Exclude general personal experience without a mentoring/support element.	P07, P12, P13 n=3; both sites; EICU/General ICU	P12: 年轻护士可能紧张或烦躁，但组长或高年资护士会介入，教经验处理。情绪上没太大变化，最多觉得工作量增加。 *Younger nurses may become nervous or frustrated, but team leaders or senior nurses step in and teach them how to manage the situation from experience.*	Supported; retained. Closely related to clinical experience but retained because transcripts described senior intervention as a social and practical resource for less experienced nurses.

Factor / type	Operational definition	Inclusion / exclusion boundary	Transcript coverage	Representative transcript evidence	Concordance decision / boundary note
**8. Subtle cues** *Facilitator*	Recognition of non-obvious behavioural or contextual cues that suggest delirium before or beyond formal screening.	Include gaze changes, purposeless movements, line pulling, sleep-wake changes, or other subtle clinical cues. Exclude overt agitation alone and formal tool results.	P02, P07, P09, P13 n=4; both sites; EICU/General ICU/RICU	P13: 首先是眼神，跟正常人不一样。......另外，他会有一些异常行为，比如手漫无目的地活动，不自觉扯身上的管道。还有半夜睡不着觉。 *The first clue may be the patient's gaze, which looks different from normal. There may also be purposeless hand movements, pulling at lines, and inability to sleep at night.*	Supported; retained. These cues were described as experiential observations and were therefore separated from both formal assessment and overt hyperactive observation.

Factor / type	Operational definition	Inclusion / exclusion boundary	Transcript coverage	Representative transcript evidence	Concordance decision / boundary note
**9. Novice inexperience**<br>*Barrier*	Reduced confidence or recognition accuracy among newer nurses because of limited exposure to delirium presentations.	Include explicit contrasts between new and experienced nurses, missed recognition, or management difficulty attributed to limited experience. Exclude general training requests without a novice-specific element.	P07, P12, P13<br>n=3; both sites; EICU/General ICU	P13: 新护士和老护士处理谵妄对比大。ICU 容易把轻病人安排给新护士，但新护士识别不了谵妄，容易夜间拔管。<br>*There is a large difference between new and experienced nurses in managing delirium. New nurses may fail to recognise delirium, which can contribute to night-time device removal.*	Supported; retained. The contrasting facilitator was senior/experienced judgement; the barrier was not treated as a universal characteristic of all junior nurses.

Factor / type	Operational definition	Inclusion / exclusion boundary	Transcript coverage	Representative transcript evidence	Concordance decision / boundary note
**10. Hypoactive-delirium knowledge gaps** *Barrier*	Explicit lack of knowledge or awareness of hypoactive delirium as a clinical subtype.	Include statements that the concept/subtype was previously unknown or poorly understood. Exclude cases where the subtype was known but difficult to detect.	P03, P07, P10, P14 n=4; both sites; EICU/General ICU/RICU	P10: 我之前不知道低活动性谵妄这个概念。我们工作中都会和患者沟通，比如巡视时会询问患者情况，能从回答和交流中发现异常。 *I did not previously know the concept of hypoactive delirium. In practice we communicate with patients during rounds and may notice abnormalities from their answers and conversation.*	Supported; retained. Separated from 'Hypoactive delirium' because this factor is a knowledge deficit rather than an observational difficulty. Retained; claim softened from “critically hindered” to “limited early recognition”.

Factor / type	Operational definition	Inclusion / exclusion boundary	Transcript coverage	Representative transcript evidence	Concordance decision / boundary note
**11. Insufficient training** *Barrier*	Perceived inadequacy of existing delirium education, especially for practical recognition and management.	Include requests for more targeted, practical, scenario-based, or clinically usable training. Exclude dissatisfaction with a specific screening instrument when training is not the issue.	P01, P04, P05, P09, P11, P12, P14 n=7; both sites; EICU/General ICU/RICU	P05: 我们有培训的 PPT，还有评分的这些让我们去做，但是我更希望可以搞个那种实战演练之类的……真的遇到谵妄病人了，你去怎么去保护自己，怎么去保护病人，怎么去给医生协助，怎么处理。 *We have training slides and scoring content, but I would prefer practical simulation—for example, when a patient actually develops delirium, how to protect ourselves and the patient, assist the doctor, and manage the situation.*	Strongly supported; retained. Many participants had received some training yet still requested practical or scenario-based preparation, supporting insufficiency rather than complete absence of education.

Opportunity: Environment and systemic constraints

Factor / type	Operational definition	Inclusion / exclusion boundary	Transcript coverage	Representative transcript evidence	Concordance decision / boundary note

Factor / type	Operational definition	Inclusion / exclusion boundary	Transcript coverage	Representative transcript evidence	Concordance decision / boundary note
**12. Adequate staffing** *Facilitator*	Sufficient personnel enabling closer observation, communication, psychological care, and non-pharmacological delirium management.	Include accounts describing how more staff or one-to-one attention would facilitate care. Exclude peer help during a single episode and high workload without explicit staffing availability.	P01, P04, P06, P09, P14 n=5; both sites; EICU/General ICU/RICU	P04: 如果人员更充足，我们能更频繁地和清醒患者交流沟通，进行一定的心理护理，可能会降低谵妄发生率，也有利于改善谵妄症状。现在人力有限，影响是明显的。 *If staffing were more adequate, we could communicate with conscious patients more often and provide psychological care, which might reduce delirium occurrence and help improve symptoms. Current staffing limits clearly affect this.*	Supported; retained. The facilitator is the availability of personnel; it was kept separate from peer support and from the barrier of staff shortage.

Factor / type	Operational definition	Inclusion / exclusion boundary	Transcript coverage	Representative transcript evidence	Concordance decision / boundary note
**13. Controlled environment** *Facilitator*	Environmental practices that reduce disturbance and support sleep, orientation, and rest.	Include dimming lights, reducing direct light/noise, using eye masks/earplugs, time orientation, or similar environmental control. Exclude descriptions of unavoidable noise/light as a barrier.	P01, P02, P04, P05, P07, P12 n=6; both sites; EICU/General ICU	P04: 我们能做的，比如关闭床头灯，避免灯光直射；让家属来时给患者准备眼罩、耳塞，尽量营造休息环境。白天也会告诉患者现在是白天、几点钟，帮助他建立时间认知。 *We can turn off bedside lights, avoid direct light, ask families to prepare eye masks and earplugs, and try to create a resting environment. During the day we also tell patients that it is daytime and what time it is to support temporal orientation.*	Supported; retained. Contrasting accounts of poorly controlled light/noise supported retaining both a facilitator (when control was feasible) and a barrier (sensory overstimulation).

Factor / type	Operational definition	Inclusion / exclusion boundary	Transcript coverage	Representative transcript evidence	Concordance decision / boundary note
**14. Single-room layouts** *Barrier*	Reduced visual access to patients when assigned beds are physically separated or distributed across rooms.	Include room separation, isolation rooms, or fragmented bed locations that make continuous observation difficult. Exclude workload caused by patient numbers without a visibility/layout component.	P01, P02, P03, P06, P14 n=5; both sites; General ICU	P14: 如果我管三个病人，他们可能分布在三个房间。如果其中一个是谵妄患者，管理难度就会大很多，因为我无法始终待在他身边。 *If I manage three patients, they may be distributed across three rooms. If one has delirium, management becomes much more difficult because I cannot remain at the bedside all the time.*	Supported; retained. Kept distinct from workload and staffing because the mechanism described was loss of visibility even when the nurse remained responsible for the same number of patients.

Factor / type	Operational definition	Inclusion / exclusion boundary	Transcript coverage	Representative transcript evidence	Concordance decision / boundary note
**15. Sensory overstimulation** *Barrier*	Noise, lighting, alarms, overnight activity, or environmental disturbance perceived to worsen rest, anxiety, orientation, or delirium.	Include 24-hour lighting, monitor alarms, voices, night-time procedures, or neighbouring activity. Exclude structural visibility problems and environmental-control strategies.	P02, P03, P04, P05, P06, P07, P09, P12, P14 n=9; both sites; EICU/General ICU/RICU	P09: 主要是灯光和噪音两个因素。ICU 基本 24 小时照明……机器报警等噪音多，会让患者紧张焦虑，再加上药物因素，可能诱发或加重谵妄。 *The main factors are light and noise. The ICU is essentially illuminated around the clock, and machine alarms can make patients tense and anxious; together with medication factors, this may trigger or worsen delirium.*	Strongly supported; retained. Some units reported better noise/light control, demonstrating contextual variation rather than a universal environmental condition.

Factor / type	Operational definition	Inclusion / exclusion boundary	Transcript coverage	Representative transcript evidence	Concordance decision / boundary note
**16. Staff shortages**<br>*Barrier*	Insufficient numbers of available staff, particularly on evening or night shifts, limiting observation and care capacity.	Include explicit references to fewer staff or insufficient personnel. Exclude competing tasks/caseload when staff number is not the stated problem.	P01, P04, P06, P09, P14<br>n=5; both sites; EICU/General ICU/RICU	P04: 中夜班人手少，通常两个人要处理医嘱、配血等工作，精力有限。<br>*Staffing is lower on evening and night shifts; two nurses may need to process orders and blood preparation, leaving limited capacity.*	Supported; retained. Separated from high workload: staff shortage concerns personnel availability, whereas workload concerns competing demands and task volume.

Factor / type	Operational definition	Inclusion / exclusion boundary	Transcript coverage	Representative transcript evidence	Concordance decision / boundary note
**17. High workload** *Barrier*	Competing patient-care demands and task volume that reduce time for sustained delirium assessment or non-pharmacological care.	Include multiple-patient caseloads, prioritising more critically ill patients, delayed communication, or insufficient time. Exclude statements solely about low staffing numbers.	P01, P02, P03, P04, P05, P06, P07, P08, P11, P12, P13, P14 n=12; both sites; EICU/General ICU/RICU	P12: 如果管 3 到 4 个病人，旁边又忙又闹，就没太多时间做非药物干预。实话实说，很多时候会优先重病人，直接请医生用药物处理，才能腾出手，这在工作量大时没法避免。 *If I am caring for three or four patients while the unit is busy, there is not much time for non-pharmacological interventions. In reality, we often prioritise the more critically ill patients and ask the doctor to use medication so that we can free up time; this can be unavoidable when workload is high.*	Strongly supported; retained. Accounts showed that workload could constrain delirium care even when participants did not explicitly attribute the problem to staffing levels.

Opportunity: Delirium management is a team sport

Factor / type	Operational definition	Inclusion / exclusion boundary	Transcript coverage	Representative transcript evidence	Concordance decision / boundary note
**18. Family engagement**<br>*Facilitator*	Family presence or communication that reassures, reorients, calms, or improves patient cooperation.	Include accounts of family presence, familiar voices, or family reassurance facilitating care. Exclude restrictions on access, which are coded separately.	P03, P04, P06, P07, P08, P09, P10, P12, P13, P14<br>n=10; both sites; EICU/General ICU/RICU	P08: 最后把家属喊来安慰、安抚，他情绪才稍微缓解一些。家属在里面陪了几个小时后，这个病人才逐渐能够接受我们的治疗和护理。<br>*After we called the family in to comfort and reassure him, his emotions eased somewhat. After a family member stayed for several hours, he gradually became able to accept our treatment and nursing care.*	Strongly supported; retained. Positive family effects were repeatedly described, while other accounts noted limits or variable effectiveness; the factor was therefore retained as a facilitator rather than an assumed universal benefit.

Factor / type	Operational definition	Inclusion / exclusion boundary	Transcript coverage	Representative transcript evidence	Concordance decision / boundary note
**19. Peer support** *Facilitator*	Immediate practical assistance from nursing colleagues during episodes of agitation, restraint, medication, or competing workload.	Include colleagues helping, redistributing work, or stepping in during acute management. Exclude baseline staffing adequacy and senior mentoring when the focus is expertise rather than extra hands.	P01, P02, P04, P05, P06, P12, P14 n=7; both sites; EICU/General ICU	P14: 如果出现谵妄患者，报告医生后，如果需要帮忙，大家都会过来。比如进行约束、用药时人手不够，同事会互相帮忙。 *When a patient develops delirium, after reporting to the doctor, everyone comes to help if assistance is needed. For example, when there are not enough hands for restraint or medication, colleagues help one another.*	Strongly supported; retained. Peer help was described as an acute social resource and was kept distinct from formal staffing levels.

Factor / type	Operational definition	Inclusion / exclusion boundary	Transcript coverage	Representative transcript evidence	Concordance decision / boundary note
**20. Physician collaboration**<br>*Facilitator*	Timely, responsive nurse-physician communication and joint management after delirium is recognised.	Include rapid medical review, treatment orders, or collaborative decision-making. Exclude delayed or wait-and-see responses, which are coded separately.	P01, P02, P04, P06, P08, P09, P10, P11, P12, P13, P14<br>n=11; both sites; EICU/General ICU/RICU	P06: 但现在配合得比较好，协作也很融洽，发现患者谵妄后，告知医生，医生会快速下达用药指令，风险也随之降低了。<br>*Collaboration is better now. When delirium is identified and the doctor is informed, medication orders are issued quickly and the perceived risk is reduced.*	Strongly supported; retained. Transcripts contained both responsive and delayed physician responses, supporting a context-dependent facilitator/barrier pair rather than a uniform pattern.

Factor / type	Operational definition	Inclusion / exclusion boundary	Transcript coverage	Representative transcript evidence	Concordance decision / boundary note
**21. Restrictive visiting policies**<br>*Barrier*	Formal limits on the timing, duration, or frequency of family access that reduce opportunities for sustained reassurance or reorientation.	Include short scheduled visits, one-person limits, or infrequent family access. Exclude general lack of family support not caused by visiting rules.	P03, P07, P09, P14 n=4; both sites; EICU/General ICU/RICU	P09: 探视制度是每天下午 3:30-4:00，每个床位每次允许 1 名家属探视，时长半小时，家属轮流进入。……我觉得帮助不大。因为探视时间太短。 *Visiting is from 3:30 to 4:00 p.m. each day, with one family member allowed per bed for half an hour. I think the benefit is limited because the visiting time is too short.*	Supported; retained. Some units allowed exceptional family access for distressed patients, indicating that restriction was policy/context dependent rather than absolute. Retained; "rigid" revised to "restrictive" in narrative.

Factor / type	Operational definition	Inclusion / exclusion boundary	Transcript coverage	Representative transcript evidence	Concordance decision / boundary note
**22. Delayed medical response** *Barrier*	Perceived delay, observation-only approach, or insufficiently prompt medical action after nurses report delirium.	Include wait-and-see responses or delayed treatment/escalation. Exclude appropriate observation based on clinical stability unless participants framed it as limiting management.	P07, P09 n=2; Site 1; EICU/RICU	P09: 大多数时候，医生知道患者出现谵妄后，更多是采取观察等待的态度，认为药物代谢完后患者自然会清醒，当然也会有使用药物干预的情况。 *Most of the time, after physicians know that the patient has developed delirium, they tend to observe and wait, assuming the patient will wake after the medication is metabolised, although medication is also used in some situations.*	Supported with limited recurrence; retained. Only a subset described this barrier, while others reported good collaboration; the variability was preserved rather than generalised. Retained; presented as variable collaboration rather than a universal pattern.

Motivation: Safety first, at any cost

Factor / type	Operational definition	Inclusion / exclusion boundary	Transcript coverage	Representative transcript evidence	Concordance decision / boundary note
**23. Professional duty** *Facilitator*	Intrinsic professional obligation to continue caring for and intervening with delirious patients despite burden or difficulty.	Include explicit statements of duty, responsibility to care, or inability to disengage from required care. Exclude fear of adverse-event consequences, coded as accountability.	P02, P07, P09, P11 n=4; Site 1; EICU/General ICU/RICU	P02: 不会。职责在那里，责任在那里，不可能不管。 *No. The duty is there and the responsibility is there; it is impossible not to intervene.*	Supported; retained. Kept distinct from accountability: professional duty reflects role obligation, whereas accountability reflects responsibility for preventable safety events.

Factor / type	Operational definition	Inclusion / exclusion boundary	Transcript coverage	Representative transcript evidence	Concordance decision / boundary note
**24. Accountability**<br>*Facilitator*	Perceived responsibility for preventing adverse events and maintaining nursing safety/quality outcomes.	Include concern that falls, line removal, or other incidents would constitute adverse events for which nursing staff are responsible. Exclude general professional duty without an adverse-event component.	P02, P05, P06, P08, P09, P11, P14<br>n=7; both sites; EICU/General ICU/RICU	P11: 会，因为如果他不小心把管道拔出来，就会酿成不良事件。<br>*Yes, because if the patient accidentally removes a line, it becomes an adverse event.*	Strongly supported; retained. The transcripts supported perceived clinical/professional accountability but did not, in this audit, establish a specific legal framework as the mechanism.

Factor / type	Operational definition	Inclusion / exclusion boundary	Transcript coverage	Representative transcript evidence	Concordance decision / boundary note
**25. Device-removal risk** *Barrier*	Fear or management burden associated with unplanned removal of airway, vascular, drainage, or other treatment devices.	Include line/tube pulling or risk of self-extubation/unplanned device removal. Exclude falls and staff injury.	P01, P02, P03, P04, P05, P06, P07, P08, P09, P11, P13, P14 n=12; both sites; EICU/General ICU/RICU	P14: 拔管，或者有些外伤、手术后的病人自己拔除引流管路，这些情况都非常危险。 *Unplanned removal of a tube, or a trauma or postoperative patient pulling out a drainage line, can be extremely dangerous.*	Strongly supported; retained. Frequently co-occurred with restraint discussions, but the factor was retained as an immediate safety threat rather than as endorsement of restraint.

Factor / type	Operational definition	Inclusion / exclusion boundary	Transcript coverage	Representative transcript evidence	Concordance decision / boundary note
**26. Falls**<br>*Barrier*	Concern that delirious or agitated patients may fall or get out of bed unsafely.	Include fall/bed-exit risk. Exclude device-removal and staff-violence risks even when mentioned in the same account.	P02, P04, P05, P06, P08, P09, P10, P13, P14<br>n=9; both sites; EICU/General ICU/RICU	P14: 紧张害怕是有的。就是担心在你无法时刻守在他身边的时候，他会不会自己拔管了，或者从床上跌下来。<br>*There is nervousness and fear. The concern is that when you cannot remain beside the patient at all times, they may remove a tube or fall from the bed.*	Strongly supported; retained. Often co-occurred with device-removal risk; separate coding was retained because participants named them as distinct adverse-event threats.

Factor / type	Operational definition	Inclusion / exclusion boundary	Transcript coverage	Representative transcript evidence	Concordance decision / boundary note
**27. Staff violence** *Barrier*	Physical aggression or injury risk directed toward nurses or other healthcare staff.	Include hitting, kicking, grabbing, head-butting, or use of objects against staff. Exclude verbal abuse without physical aggression.	P01, P04, P05, P06, P07, P08, P09, P10, P12, P13, P14 n=11; both sites; EICU/General ICU/RICU	P06: 有一个特别壮实的病人，出现攻击行为时，用头撞击我们同事的头部，当时就把同事撞晕了。 *There was a particularly strong patient who, during an aggressive episode, struck a colleague's head with his own and knocked the colleague unconscious.*	Strongly supported; retained. Accounts ranged from threat to actual injury; the factor was retained as occupational safety risk and kept separate from verbal abuse. Retained; wording changed from "profound" and "traumatic" to cautious description.

Motivation: Weathering the emotional storm

Factor / type	Operational definition	Inclusion / exclusion boundary	Transcript coverage	Representative transcript evidence	Concordance decision / boundary note
**28. Cognitive empathy** *Facilitator*	Reframing aggressive or difficult behaviour as illness-related and not intentionally hostile.	Include statements that patients 'did not mean it', could not control behaviour, or should be understood because of illness. Exclude emotional detachment without illness-based reinterpretation.	P01, P05, P06, P07, P08, P09, P10, P11, P12, P13 n=10; both sites; EICU/General ICU/RICU	P10: 他肯定不是故意的，就是用力薅我，还踢过我一下。后来我们对他进行了约束，之后就没什么问题了，毕竟是患者，能理解。 *He certainly did not do it intentionally; he grabbed me hard and kicked me once. We later restrained him and the situation settled. After all, he was a patient, and I could understand that.*	Strongly supported; retained. Empathy coexisted with distress in several accounts; it was therefore treated as a coping facilitator rather than evidence that emotional burden was absent.

Factor / type	Operational definition	Inclusion / exclusion boundary	Transcript coverage	Representative transcript evidence	Concordance decision / boundary note
**29. Emotional boundaries**<br>*Facilitator*	Deliberate limits on emotional or relational involvement while still meeting reasonable patient needs.	Include explicit statements about setting limits/boundaries in care. Exclude simply leaving work concerns after handover.	P04<br>n=1; Site 1; EICU	P04: 对谵妄患者主要还是两点：一是预防非计划性拔管等风险；二是在允许范围内尽可能满足患者需求，但也要有界限。<br>*For patients with delirium, there are two main points: preventing risks such as unplanned device removal, and meeting patients' needs as far as possible within what is permitted, while still maintaining boundaries.*	Supported with limited recurrence; retained. This was a specific strategy described by one participant and was retained as a minor facilitator without implying broad prevalence.

Factor / type	Operational definition	Inclusion / exclusion boundary	Transcript coverage	Representative transcript evidence	Concordance decision / boundary note
**30. Off-duty disengagement** *Facilitator*	Intentional psychological separation from work-related distress after handover or off duty.	Include trying not to think about the case after work, handing over responsibility, or focusing on non-work activities. Exclude avoidance of care during the shift.	P03, P11 n=2; Site 1; General ICU/RICU	P03: 如果确实没有办法干预，确实持续烦躁的话，那就只能是下了班之后就忘掉了。就交班交出去了，就是最好的一个解决方式，就赶紧下班。 *If there is truly no effective way to intervene and the agitation continues, the only thing is to forget it after work. Once the handover is complete, that is the best solution—just finish the shift and leave.*	Supported with limited recurrence; retained. The accounts framed disengagement as recovery between shifts, not withdrawal from professional responsibilities while on duty. Retained; " ultimate self-preservation mechanism" softened to recovery between shifts.

Factor / type	Operational definition	Inclusion / exclusion boundary	Transcript coverage	Representative transcript evidence	Concordance decision / boundary note
**31. Emotional toll** *Barrier*	General mental and physical exhaustion, frustration, anxiety, or emotional burden associated with delirium care.	Include broad fatigue, irritability, psychological strain, or burden. Exclude the more specific experiences of helplessness and verbal abuse when those are the primary meaning.	P01, P02, P03, P04, P05, P06, P07, P08, P09, P11, P13, P14 n=12; both sites; EICU/General ICU/RICU	P09: 只要有谵妄患者，尤其是值夜班时，会感到心力交瘁。 *Whenever there is a patient with delirium, especially on a night shift, it can feel mentally and physically exhausting.*	Strongly supported; retained. Broad emotional burden was recurrent, while more specific emotions were retained as separate factors to avoid collapsing distinct experiences.

Factor / type	Operational definition	Inclusion / exclusion boundary	Transcript coverage	Representative transcript evidence	Concordance decision / boundary note
**32. Helplessness**<br>*Barrier*	Feeling unable to change the patient's situation or provide the desired solution because key determinants lie outside nursing control.	Include inability to facilitate transfer, resolve underlying conditions, or meaningfully change the situation. Exclude general tiredness or frustration caused by workload alone.	P02, P07, P09<br>n=3; Site 1; EICU/General ICU/RICU	P02: 有的患者想转科，但这不是我能完全干预的；再加上原发疾病限制，不能转科时，我更干预不了，所以会觉得很无力。<br>*Some patients want to transfer out, but that is not something I can fully control; when the underlying illness prevents transfer, there is even less I can do, so I feel powerless.*	Supported; retained. Kept separate from emotional toll because the defining feature was perceived lack of control rather than exhaustion.

Factor / type	Operational definition	Inclusion / exclusion boundary	Transcript coverage	Representative transcript evidence	Concordance decision / boundary note
**33. Verbal abuse** *Barrier*	Emotional distress caused specifically by insulting, abusive, or demeaning speech from delirious patients.	Include accounts of being cursed at or verbally insulted and the associated discomfort, hurt, or sense of grievance. Exclude physical aggression without abusive language.	P05, P06, P07, P09, P12 n=5; both sites; EICU/General ICU/RICU	P09: 我们遇到过很多谵妄患者，最难以接受的是他们对医务人员的辱骂。虽然知道患者是无心的，但听到后还是会心里不舒服、感到委屈。 *We have encountered many patients with delirium, and the most difficult aspect is their verbal abuse toward healthcare staff. Although we know the patient does not intend it, hearing it still causes discomfort and a sense of being wronged.*	Supported; retained. Participants could cognitively understand the behaviour as illness-related while still feeling hurt, supporting a distinct factor alongside cognitive empathy.

4. Audit interpretation and use in revision

The audit supports retention of the six-theme, 33-factor structure as a transparent representation of the fixed analytic framework. For P02, P04, P09, P12, and P14, participant-level coverage in this document reflects the final YC–YG consensus reported in Supplementary File S2; coverage for the remaining nine transcripts reflects the full-dataset revision-stage audit. The most important strengthening is not numerical recurrence but clearer factor boundaries: for example, staff shortages were separated from high workload; professional duty from accountability; hypoactive-delirium knowledge gaps from difficulty detecting hypoactive delirium; and physician collaboration from delayed medical response.

Contrasting accounts were retained rather than removed. Examples include variable experiences of CAM-ICU usability, units with relatively controlled versus overstimulating environments, and both responsive and delayed physician collaboration. These contrasts should be used in the manuscript to avoid universal claims and to demonstrate consideration of divergent evidence.
